# Dental transposition of canine and lateral incisor and impacted central incisor treatment: A case report

**DOI:** 10.1590/2176-9451.19.1.106-112.oar

**Published:** 2014

**Authors:** Tarcisio Jacinto Gebert, Vinícius Canavarros Palma, Alvaro Henrique Borges, Luiz Evaristo Ricci Volpato

**Affiliations:** 1 Masters student, Integrated Dental Sciences Program, University of Cuiabá (UNIC).; 2 PhD in Dentistry, State University of São Paulo (UNESP). Professor, Integrated Dental Sciences Masters Program, UNIC.; 3 PhD in Dentistry, University of Ribeirão Preto (UNAERP). Professor, Integrated Dental Sciences Masters Program, UNIC.; 4 PhD in Dentistry, School of Dentistry - USP/ Bauru. Professor, Integrated Dental Sciences Masters Program, UNIC.

**Keywords:** Impacted tooth, Ectopic tooth eruption, Corrective orthodontics

## Abstract

**Introduction:**

Dental transposition and impaction are disorders related to ectopic eruption or failure in tooth eruption, which can affect child physical, mental and social development and may be difficult to be clinically solved.

**Methods:**

We describe a case of transposition between the upper left canine and lateral incisor associated with impaction of the central incisor on the same side, in a 12-year-old patient. Conservative treatment involving surgical-orthodontic correction of transposed teeth and traction of the central incisor was conducted.

**Conclusion:**

The option of correcting transposition and orthodontic traction by means of the segmented arch technique with devices such as cantilever and TMA rectangular wire loops, although a complex alternative, was proved to be esthetically and functionally effective.

## Abstract (pt)

**Introdução:**

transposição e impacção dentárias são distúrbios relacionados à erupção ectópica ou à falha na erupção dentária, que podem afetar o desenvolvimento físico, psíquico e social da criança, e que podem ser de difícil resolução clínica.

**Métodos:**

é descrito um caso clínico de transposição entre o canino e o incisivo lateral superior esquerdo, associado à impacção do incisivo superior, do mesmo lado, em uma paciente de 12 anos de idade. Optou-se pela realização de tratamento ortodôntico-cirúrgico conservador, envolvendo a correção ortodôntica dos dentes transpostos e o tracionamento do incisivo central impactado.

**Conclusão:**

a opção pela correção da transposição e tracionamento ortodôntico com a utilização da técnica do arco segmentado com uso de dispositivos como cantiléver e alças em fios TMA retangulares, apesar de ser uma alternativa de execução complexa, mostrou-se efetiva do ponto de vista estético e funcional.

## INTRODUCTION

Impaction is a condition in which complete tooth eruption is hampered by contact with another tooth.^1^ It is characterized by dental absence in the arch after its usual period of eruption,^2^ and its etiology may be related to general or local factors.^3^ General factors include endocrine disorders, febrile diseases, radiation, heredity and development factors that may alter the eruption trajectory of the tooth germ.^4^ Local causes include lack of space in the arch, trauma, blocking by supernumerary tooth and lack of coordination between the formation of permanent teeth and deciduous exfoliation.^5^

Impaction is twice as common in females^6^ and can occur with any teeth, but the most affected are the lower third molars, upper canines and upper third molars, upper and lower second premolars and upper central incisor.^7^ When the upper permanent incisors are impacted, there may be impairment in physical, psychological and social development of the child.^8^ After diagnosis, the therapeutic decision should prioritize tooth eruption.^9^

Conversely, tooth transposition, reported since the early nineteenth century, is described not only as a reversal of position between two teeth in the same quadrant of the dental arch, especially in relation to their roots; but also as the development or eruption of a tooth in a position normally occupied by a non-adjacent element.^10^ It is considered real or complete when the tooth is in fully exchanged position in the dental arch and its roots are parallel to the other teeth; and incomplete when the teeth involved are not in fully exchanged position.^11,12,13^ It is also more prevalent in females, in the upper arch, and of unilateral type.^11,12,14^ The upper permanent canine is the most involved tooth, transposing with the first premolar in 80% of cases and with the lateral incisor in 20%.^12^ The causes of tooth transposition also involves general or local factors such as genetic factors with multifactorial causes of inheritance, dental anomalies (congenital absence of the lateral incisor, cone-shaped lateral incisor, rotations and hypodontia), migration of the developing tooth from its normal eruption path, root dilacerations, dental trauma and intervention in the development of the dental lamina.^15^

Early diagnosis of transposition in tooth development and impaction is essential, and greatly influences the prognosis.^16^ Depending on the exact position of the impacted incisor, orthodontic movement and positioning in the dental arch can vary widely. During orthodontic mechanics, occlusal interference should be avoided and root resorption should be controlled by periapical radiography so that bone loss does not occur, specially in the buccal bone plate.^15^ Incisors that are horizontally inclined, in severe ectopic position or in real or complete tooth transposition are more difficult to treat. In cases of late diagnosis, orthodontic planning interferes not only in the decision of extracting impacted or transposed teeth, but also in the correction of the order of tooth position.^11,12,15^ Mechanical traction is preferred in the upper central incisors, but potential esthetic and periodontal problems may arise.^17^

Imaging tests are important aids in the diagnosis of these complications. They include the use of periapical, occlusal and panoramic radiographs or cephalograms,^18^ and, most recently, cone beam tomography.^23^

The objective of this paper is to report the treatment of a patient with tooth transposition between canine and lateral incisor of the upper left side associated with impaction of the upper central incisor on the same side.

## CASE REPORT

A 12-year-old, female, Caucasian patient arrived for evaluation accompanied by her guardian, with her initial documentation at hand and orthodontic appliance installed. She had been undergoing treatment for 2 years and 7 months. Her chief complaint was that her front tooth was not born and two teeth had been born in exchanged position. The person responsible for the patient presented a transfer letter in which the orthodontist suggested clinical crown recontouring of the upper left lateral incisor into canine and upper left canine into lateral incisor, in addition to extraction of the retained central incisor.

**Figure 1:**
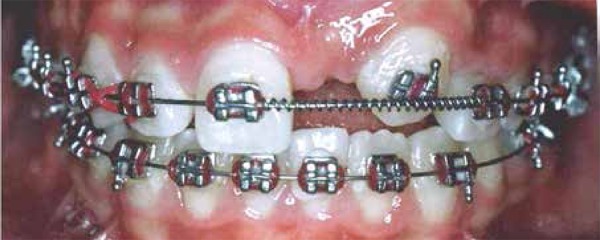
Patient's initial photograph showing fixed appliance set with transposition between the upper left canine and lateral incisor, and the absence of upper central incisor on the same side.

Clinical examination revealed that the patient had permanent dentition with Angle Class II malocclusion on the left side due to migration of the posterior superior segment to a more anterior position; Class I malocclusion on the right side; upper midline deviation to the left; absence of tooth #21 with bulging in the vestibular region just below the anterior nasal spine (ANS), lack of space for its eruption, and transposition between teeth #22 and 23 (Fig 1). New radiographic documentation was, then, requested.

**Figure 2:**
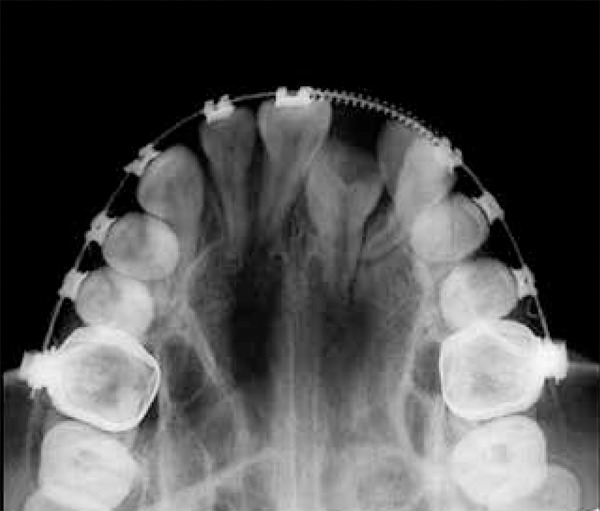
Occlusal radiograph revealing impaction of tooth #21 with complete root formation.

**Figure 3:**
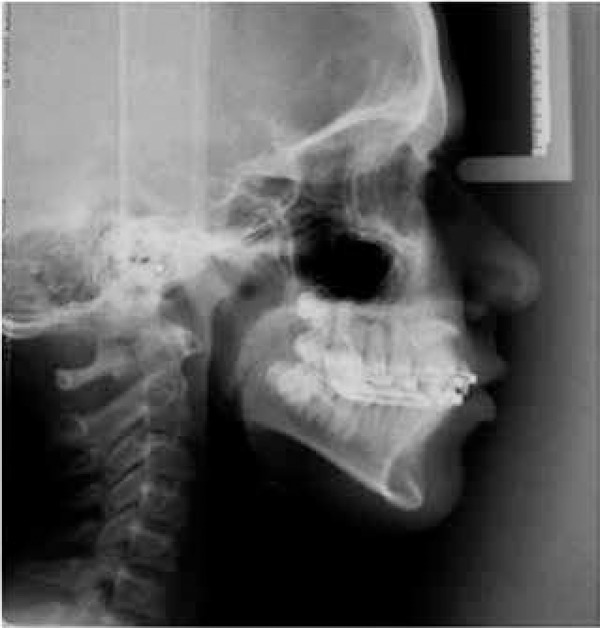
Lateral cephalogram revealing impaction of tooth #21 with coronal long axis horizontally positioned between the middle and apical third of adjacent teeth roots.

**Figure 4:**
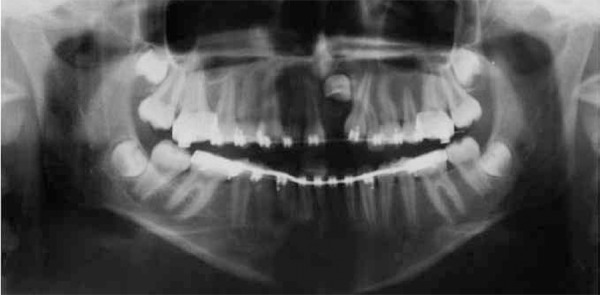
Panoramic radiograph revealing impaction of tooth #21, #22 and #23 with incomplete transposition.

Panoramic, occlusal and periapical radiographs as well as cephalograms revealed impaction of tooth #21. Its root was fully formed (Fig 2) and had the coronal long axis in a more horizontal position between the middle and apical third of the adjacent teeth roots (Fig 3). Incomplete transposition between teeth #22 and 23 and image overlap of tooth #22 root apex and tooth #21 root were confirmed (Fig 4).

Once diagnosis had been completed, orthodontic-surgical conservative treatment was planned, although impaction of tooth #21 and transposition of teeth #22 and 23 represented unfavorable factors. Treatment plan included an attempt to correct transposition of teeth #22 and 23 and traction of tooth #21, which was accepted by the patient and her guardians.

**Figure 5:**
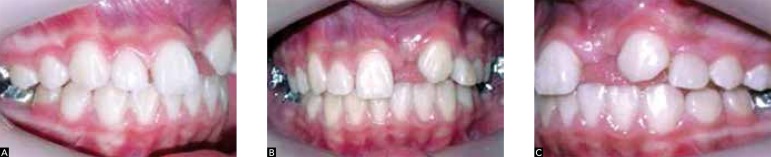
Intraoral photographs with fixed appliance removed and new bands with triple tubes properly cemented. A) Right lateral view. B) Frontal view. C) Left lateral view.

**Figure 6:**
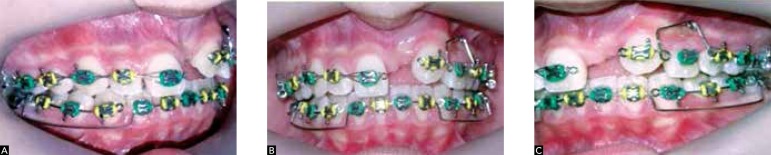
Intraoral photograph showing fixed appliance: A) Right lateral view. B) Frontal view and C) Left lateral view with TMA semiarch for #23 intrusion.

**Figure 7:**
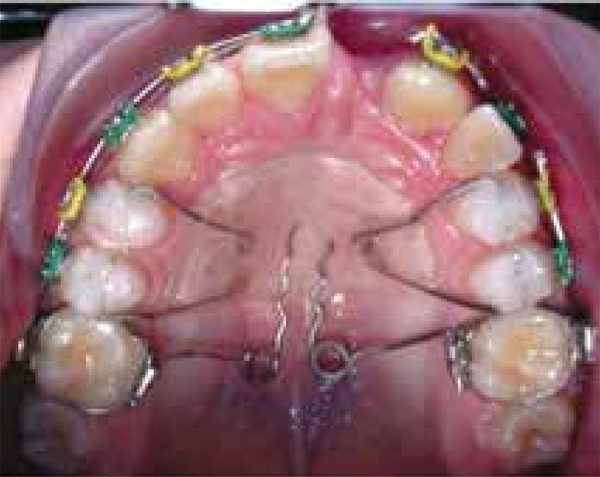
Hilgers appliance properly installed.

**Figure 8:**
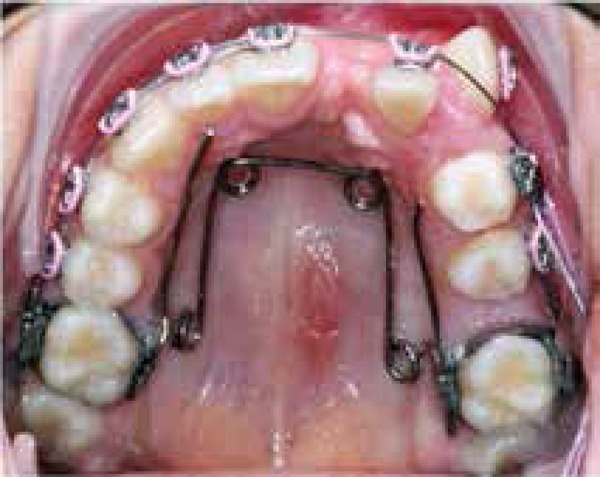
Quad-helix appliance installed to maintain distalization of #26.

The orthodontic procedures began by removing the appliance previously installed on both arches of the patient, since it was an appliance aimed at a different purpose. After removal and prophylaxis, new bands and weld of upper triple tube and lower double tube were prepared, in addition to cementing them on the upper and lower first molars (Fig 5). The upper arch was shaped with impression material for the fabrication of Hilgers auxiliary appliance. Thereafter, the fixed metal orthodontic appliance was set with 0.018 x 0.030-in slot in both arches, semi-arches of stabilization with blue 0.016 x 0.016-in Elgiloy wire on the lower right and left sides as well as on the upper right side, an utility arch (UA) in the lower arch, and, on the upper left side, a semi-arch with helicals for intrusion of tooth #13 with TMA 0.017 x 0.025-in wire, with intrusive force of 45 grams to attempt dental transposition (Fig 6). After two days, the Hilgers appliance was installed for upper left molar distalization and anchorage of the upper premolars on the same side, thus, avoiding extrusion (Fig 7). After eight months, the necessary upper molar distalization and canine intrusion were obtained. The Hilgers appliance was removed and a quad-helix appliance (QH) was installed with 0.90 mm stainless steel wire to anchor and maintain upper left molar distalization (Fig 8). Additionally, 0.014-in round nickel-titanium alloy (NiTi) archwire was installed for teeth alignment and leveling without inclusion of tooth #23.

**Figure 9:**
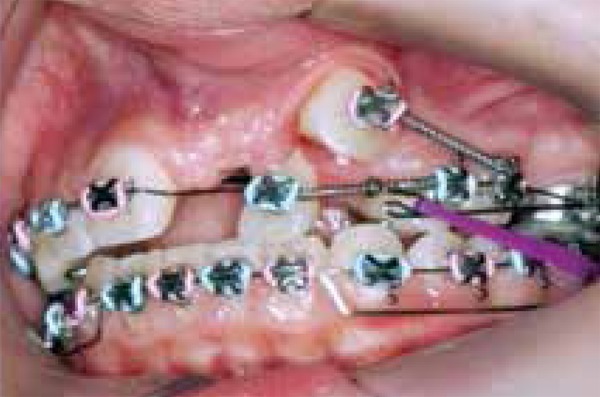
Distalization of tooth #23 with closed NiTi spring and mesialization of #22 with open NiTi spring.

**Figure 10:**
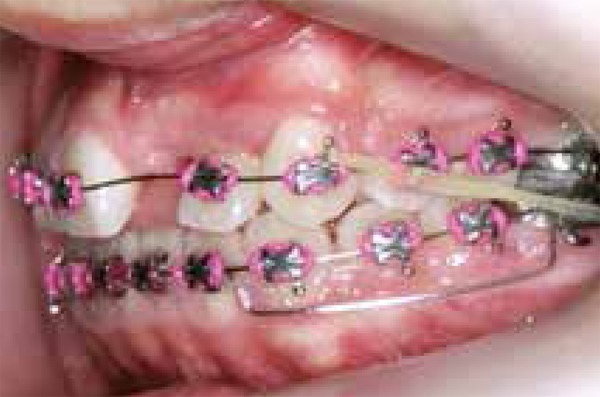
Upper arch alignment after transposition.

**Figure 11:**
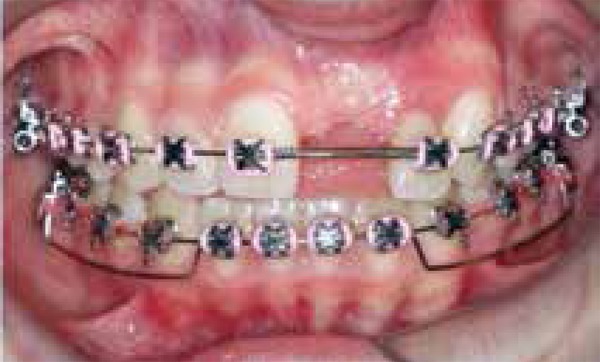
Upper arch with open steel spring used to keep the space left for traction of retained tooth #21.

Cortical anchorage was performed in the lower arch with UA, raise of bite with compound resin in the occlusal region of the lower molars and, after alignment and leveling, a 0.014-in stainless steel wire was installed. Then, mesialization of the upper left lateral incisor began to transpose it with the upper left canine by means of an open nickel-titanium (NiTi) spring in the stainless steel wire between the upper left first premolar and upper left lateral incisor. A closed nickel-titanium spring 50g/f was placed from the upper left second premolar to the upper left canine for canine distalization. A semi-arch with rectangular 0.017 x 0.025-in stainless steel wire with 3/16-in medium elastic was also installed (Fig 9). After transposition was completed, alignment and leveling were performed with 0.014-in NiTi wire (Fig 10) up to the 0.016 x 0.016-in square stainless steel wire for better stabilization. An open steel spring was used between teeth #11 and 22 to keep the recovered space that would be occupied by tooth #21 (Fig 11). Afterwards, surgical exposure and bonding of the traction accessory were performed.

**Figure 12:**
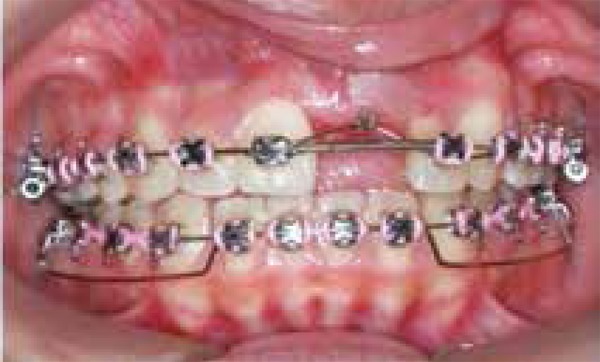
Traction of tooth #21 carried out with 0.014?/span> NiTi wire superimposed to 0.016 x 0.016-in square stainless steel archwire with open spring between #11 and #22.

Closed flap was the surgical technique chosen for traction. After 10 days, the sutures were removed and traction of tooth #21 began with 0.014-in NiTi wire superimposed to the square stainless steel archwire with open spring, by applying low magnitude forces in order to prevent unwanted movement of the adjacent teeth (Fig 12). After eruption of the retained tooth, the button that was placed during surgery was removed and the bracket was positioned. New alignment and leveling were performed by inserting the tooth in the arch, starting with 0.014-in NiTi wire up to the 0.016 x 0.016-in steel square wire during a period of 9 months.

**Figure 13:**
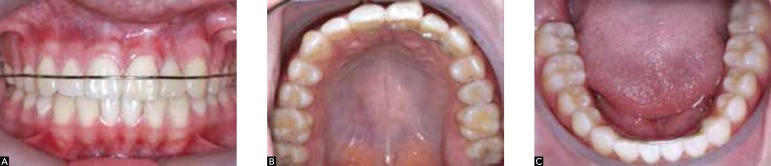
Final intraoral photographs: A) Frontal view with Hawley retainer. B) Occlusal view of upper arch with fixed retainer from tooth #11 to 23. C) Lower occlusal view with fixed lingual arch.

**Figure 14:**
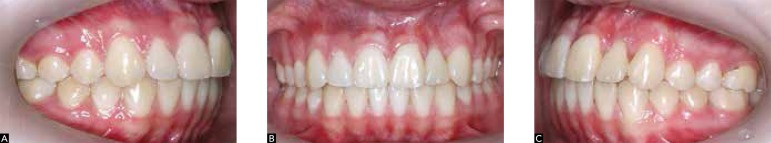
Final intraoral photographs: A) Right lateral view. B) Frontal view. C) Left lateral view.

**Figure 15:**
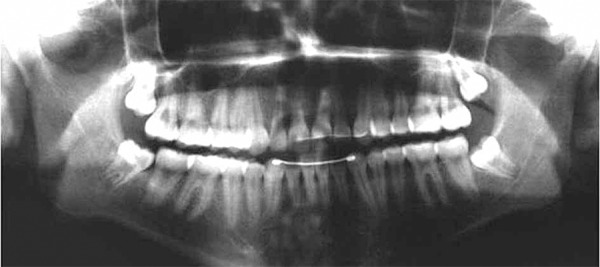
Final panoramic radiograph.

After thirty-nine months of active orthodontic treatment, the fixed orthodontic appliance was removed and the retainer was installed. For the upper dental arch, a fixed retainer was fabricated with 0.015-in Twist-flex wire from the left canine to the right central incisor, associated with a removable plate with continuous arch adapted to the buccal surfaces, from second molar to second molar, without occlusal interferences. As for the lower dental arch, a fixed lingual arch fabricated with 0.80 mm stainless steel wire was adhered to the lingual surfaces of lower canines, with light-cured resin (Fig 13). The case has been finalized and it is currently under clinical (Fig 14) and radiographic follow-up (Fig 15).

## DISCUSSION

Tooth transposition is classified into complete and incomplete according to the position of the crowns, roots and apex of transposed teeth. In incomplete transposition, the apexes tend to remain in their original position, while only the crowns suffer transposition.^11,12,13^

The basal bone most often affected is the maxilla, with higher incidence of unilateral transposition, which, in this case, the left side prevailed.^12-24^ The teeth mostly affected by transposition are canine and first premolar^12^ as well as canine and lateral incisor.^25^ The case reported herein presented incomplete transposition between the canine and lateral incisor on the left side, associated with impacted upper left central incisor.

In the presence of an impacted tooth, a frequent complication of traction is the possibility of the tooth not moving due to ankylosis.^26^ Moving an impacted tooth involves risks of devitalization, discoloration, external root resorption, injury to adjacent teeth, alveolar bone loss, gingival recession, increase in clinical crown length and tooth sensitivity, which can lead to esthetic problems or tooth loss.^27^ In the reported case, alveolar bone resorption existed prior to treatment. Gingival recession and increased clinical crown were also observed.

Traction and conservation of retained anterior teeth, both esthetically and functionally, it is the alternative therapy with the most favorable outcomes.^28^ There is a great demand for satisfactory esthetic outcomes in the anterior region, and no prosthetic material is superior to the tooth itself. Occlusal problems also decrease, since there is no tooth loss and the arch remains symmetrical and complete.^8^ Another important aspect is that the volume of alveolar bone loss resulting from extraction of the incisor is avoided, a frequent situation which is difficult to be solved.^28^ Thus, in the case reported herein, traction of tooth #21 was the treatment of choice, yielding satisfactory esthetic and functional results. The surgical technique used was the closed technique, in which the tooth is tractioned inside the mucosa and alveolar bone. This technique presents stability, periodontal anatomy and final esthetic results that are more favorable than apical reposition of the flap with immediate exposure of the crown after surgery.^28^

The technique as well as the orthodontic appliances used in the traction of impacted teeth or in transposition correction will depend on a correct diagnosis and treatment plan.^1,3,7,15^ When adjacent teeth require individualized and controlled movements, fixed appliances are indicated^8^. The mechanics of choice must be carefully planned.^3,7,13,15^ In this case, the segmented arch technique was performed with the use of devices such as cantilever and loop in rectangular wires, which allowed the professional to work with a control system of strength with regard to movement of the central incisor, lateral incisor and canine as well as anchorage units performed individually.^3,7,10^ However, the treatment involves risks, requiring extremely controlled mechanics, care and accurate application to overcome the possibility of failure.

Maintaining the central incisor, lateral incisor and canine in their usual position was essential to achieve balanced occlusion, periodontal health, facial harmony and for establishing patient's esthetics. Canine guidance was another very positive aspect of the case. As a consequence, protrusive and lateral movements were properly maintained, constituting an element of protection of the stomatognathic system, as well as the molar ratio of Class I Angle and correct overjet and overbite, thus allowing occlusal stability and proper dental esthetics.^3,7^

In view of a case involving tooth transposition between canine and lateral incisor associated with impaction of the central incisor, the orthodontist must be committed to positioning these teeth correctly, leveling and aligning them in the dental arch within the biological principles that guide the integrity of adjacent tissue structures, thus resulting in a successful treatment.^8,10,12^ The technique of traction and tooth transposition proved to be highly satisfactory, restoring patient's esthetics and harmonious occlusal relationships.

## CONCLUSION

In the reported case, the choice of transposition and orthodontic traction carried out by means of the segmented arch technique performed with devices such as cantilever and loops in rectangular wires, despite being a more difficult alternative, proved to be effective from an esthetic and functional standpoint.
